# Dopamine Agonists for Pituitary Adenomas

**DOI:** 10.3389/fendo.2018.00469

**Published:** 2018-08-21

**Authors:** Odelia Cooper, Yona Greenman

**Affiliations:** ^1^Pituitary Center, Cedars-Sinai Medical Center, Los Angeles, CA, United States; ^2^Institute of Endocrinology, Metabolism and Hypertension, Tel Aviv-Sourasky Medical Center, Sackler Faculty of Medicine, Tel Aviv University, Tel Aviv, Israel

**Keywords:** dopamine agonist, pituitary adenoma, nonfunctioning pituitary tumor, Cushing's disease, acromegaly

## Abstract

Dopamine agonists (DA) are well established as first-line therapy for prolactinomas. These tumors express high levels of dopamine 2 receptors (D2R), leading to the strong efficacy of DA in reducing tumor size and hormonal secretion. Other pituitary tumor subtypes express D2R to varying degrees, leading to an extensive body of research into potential off-label use of DA in non-prolactinoma pituitary tumors. Preclinical models of Cushing's disease, acromegaly, and nonfunctioning pituitary tumors (NFPT) demonstrate D2R expression in cell lines and cultured tumors as well as effectiveness of DA in reducing hormonal secretion in functioning tumors and arresting tumor proliferation. Clinical studies have shown some efficacy of DA in treatment of these tumors. In Cushing's disease, DA therapy results in normalization of urinary cortisol levels in approximately 25% of patients, but reported rates of tumor shrinkage are very low; in acromegaly, DA therapy leads to normalization of insulin-like growth factor I and tumor shrinkage in approximately one-third of patients, and improved responses when used in combination with somatostatin receptor ligands. Among patients with NFPT, pooled results show 30% experience reduction of tumor size and 58% show stabilization of disease. DA therapy appears to have some clinical benefit in patients with non-prolactinoma pituitary tumors, and may be an option for medical therapy in some clinical scenarios.

## Introduction

Dopamine modulates pituitary hormone secretion through the dopamine subtype 2 receptor (D2R). Dopamine binding to D2R in pituitary cells activates signaling cascades, leading to adenylyl cyclase and phosphatidylinositol metabolism inhibition, potassium channel activation, and reduced L-type and T-type calcium currents (1). Dopamine agonists (DA) targeting D2R yield excellent therapeutic response in prolactinomas and are the established first-line treatment (2).

D2R is mainly expressed in lactotrophs, but has been localized to all anterior pituitary cell types (3), leading to off-label evaluation of cabergoline, bromocriptine, and quinagolide as DA treatment in other pituitary tumors. This review focuses on the use of DA in Cushing's disease (CD), acromegaly, and nonfunctioning pituitary tumors (NFPT). Although many studies explored measurements of D2R expression as a predictor of response to DA therapy, this remains primarily a research tool. In current clinical practice, D2R expression is likely limited as a predictor of response to DA therapy.

## Cushing's disease

### Preclinical studies

Murine and cell-line studies indicate D2R is a potential target in CD. The intermediate lobe of the pituitary in rodents is under tonic inhibition by hypothalamic dopaminergic neurons acting through D2R (4), and is hypertrophied in D2R-deficient mice, exhibiting increased expression of adrenocorticotropic hormone (ACTH) and its precursor proopiomelanocortin (POMC). Bromocriptine inhibited proliferation and induced apoptosis of mouse pituitary corticotroph ACTH-secreting AtT20 cells (5, 6). Similarly, cabergoline treatment of cultured tumors in canine CD moderately expressing D2R mRNA attenuated ACTH secretion (7).

Up to 75% of human corticotroph adenomas express D2R (8, 9). In 15 of 20 ACTH-secreting adenomas, D2R immunostaining was moderate or strong, and D2R mRNA was detected in 10 of 12 cases. DR2-expressing primary tumor cell cultures showed dose-dependent ACTH inhibition with bromocriptine and cabergoline, whereas tumors not expressing D2R did not respond (8).

It is unclear whether corticotroph tumor features affect D2R expression. D2R mRNA was detected in 5 of 8 ACTH-secreting tumors and in 6 of 8 silent corticotroph adenomas (10), suggesting D2R expression may be seen across all corticotroph subtypes. However, correlation between D2R mRNA and protein expression in this study was poor. Others found lower D2R mRNA expression levels in overt or subclinical CD adenomas vs. silent ACTH-producing tumors (11), as well as higher expression levels in noninvasive microadenomas vs. higher-grade adenomas (12). Although data are limited, it is possible that such tumor features influence D2R expression levels, potentially affecting DA effectiveness.

### Clinical efficacy

Cabergoline has been studied as primary therapy for CD and in patients with relapsed or recurrent disease. In a review of 5 studies including 88 patients followed for 3–60 months, 25–40% of cases saw remission, with a subsequent escape rate of 16% (13). Pooled results of published series show that 25% of patients respond with normalization of 24–h urinary free cortisol (UFC) secretion (Table 1).

**Table 1:** Response to dopamine agonists in Cushing's disease.

**Study (Study type)**	***N***	**Treatment duration**	**Tumor shrinkage (*n*)**	**UFC response**	**ACTH response**
**CAB TREATMENT**					
Pivonello et al. (8) (retrospective)	10	3 months	N/A	4 complete response; 2 decreased	6 decreased
Pivonello et al. (14) (prospective)	20	3–24 months	4	Short term: 7/15 complete, 8/15 partial response Long term: 8 controlled	N/A
Godbout et al. (15) (retrospective)	30	20–28 months	1	11 (36%) complete, 4 partial response	N/A
Lila et al. (16) (prospective)	20	12 months	N/A	5 complete (serum cortisol)	No change
Vilar et al. (17) (prospective)	12	6 months	N/A	3 complete 6 partial response (>25% decrease)	N/A
Barbot et al. (18) (prospective)	6	12 months	N/A	2 complete, 1 partial response	No change
Burman et al. (19) (prospective)	24	1.5 months	N/A	2 complete, 3 partial response (>50% decrease)	6 decreased
Ferriere et al. (20) (retrospective)	53	3–105 months	N/A	21 complete, 4 partial response Long term: 12 complete response	Decreased in complete responders
Total for CAB	175		5/50 (10%)	56/175 (32%) complete response 28/175 (16%) decreased	
**BRC TREATMENT**					
Kennedy et al. (21)	5	16–87 weeks		No change	4 decreased
Lamberts et al. (22)	13	One dose		6 decreased	6 decreased
Boscaro et al. (23)	6	2 days			2 decreased
Koppeschaar et al. (24)	13	One dose		3 decreased (serum cortisol)	
Mercado-Asis et al. (25)	6	Up to 36 months	1	1 complete response, 3 decreased	
Invitti et al. (26)	6	3 months	1	No change	No change
Total for BRC	49		2/49 (4%)	1/49 (2%) complete response 12/49 (24%) decreased	
Total overall	224		7/99 (7%)	57/224 (25%) complete response 40/224 (18%) decreased	

*Case series ≥5 patients and clinical studies included*.

*ACTH, adrenocorticotropic hormone; BRC, bromocriptine; CAB, cabergoline; N/A, data not available; UFC, urinary free cortisol*.

Patients who respond to DA therapy may achieve complete (defined as normalized UFC) or partial response (variably defined as 25% or 50% decrease in UFC from baseline, or < 125% ULN) by 3–6 months after treatment initiation (8, 14, 15, 19, 27). Dose escalation may enhance efficacy in patients who initially achieved partial response, but escape may occur within < 2 years (14, 15, 17).

Pivonello et al. treated 10 CD patients for 3 months with escalating doses of cabergoline starting at 1 mg/week and increasing 1 mg/week until UFC normalization was achieved; 4 had a complete response and 2 had a partial response (8). Extending treatment in an additional 10 patients yielded response in 15 of 20 subjects by month 3, with 6 of 8 partial responders achieving UFC normalization after 6–12 months. Of note, 2 complete responders and 3 partial responders escaped treatment after 12–18 months. At 12 months, 10 subjects were controlled on a median cabergoline dose of 6 mg/week; at 24 months, only 8 were still controlled and overall efficacy was calculated at 40% (14).

In a larger study of 30 patients with CD, 27 of whom had relapsed/recurrent disease, Godbout et al. reported 11 complete responders and 4 partial responders with a mean cabergoline dose of 1.5 mg/week and an average time to normalization of 4.2 months. Nine of 11 complete responders maintained eucortisolemia while on treatment for a mean of 37 months, for an overall response rate of 30%; partial responders saw UFC increase after 6 months (15).

Finally, Ferriere et al. reported on a multicenter retrospective study of 53 CD patients, 44 of whom had relapsed/recurrent disease, who were treated with a mean cabergoline dose of 2.5 mg/week for a median of 7 months. In the first year, 21 patients (40%) achieved complete response and 4 achieved partial response; mean cabergoline dose in responders was 1.5 mg/week (range, 0.5–4.0 mg/week). After more than 1 year, 7 of 18 relapsed and required increased treatment doses; 3 achieved secondary UFC normalization. Overall response rate was 23% at 32.5 months (20).

Metabolic symptoms typically improve in responders (14, 20). Tumor size can decrease or remain stable (14) but residual tumor may be undetectable on repeat MRI; changes in tumor size may correlate with UFC response (15). However, reported rates of tumor shrinkage across studies remain low (Table 1).

Tumor features may not be useful in predicting response to cabergoline. In Pivonello's original cohort, all 6 of 10 who achieved complete or partial response showed D2R mRNA expression (8), but van der Pas et al. saw no differences in D2R mRNA or protein expression in their cohort of 33 (28). D2R isoform expression may be more useful. In prolactinomas, the short isoform (D2S) regulates prolactin (PRL) synthesis and lactotroph proliferation while the long isoform (D2L) regulates PRL release (29). Consistent with the association between D2L overexpression and DA resistance (29, 30), the one nonresponder in Pivonello's study who expressed D2R harbored the long D2L isoform while 3 complete responders expressed D2S (8).

Given the low success rate of DA monotherapy in CD, Barbot et al. added ketoconazole in an attempt to improve therapeutic response. Fourteen patients with *de novo* or persistent/recurrent CD randomly assigned to cabergoline or ketoconazole monotherapy for 6 months were switched to combination therapy for 6–12 months if UFC and late-night salivary cortisol (LNSC) did not normalize despite escalated monotherapy doses. Complete response rate was 33% with cabergoline monotherapy and 63% with ketoconazole monotherapy; with the combination, 79% achieved UFC normalization and clinical response persisted for at least 6 months (18). Those with persistent/recurrent disease were more likely to respond.

Pregnancy in women with CD is uncommon, but is associated with significant maternal and fetal morbidity (31). Surgical resection of the adenoma is the recommended treatment approach; steroidogenesis inhibitors are either contraindicated or indicated only if the risk to the fetus is outweighed by the risks of nontreatment (32). Case reports suggest that cabergoline 2 mg/week to 5 mg/week might be an option for pregnant women with recurrent disease (33, 34), with patients demonstrating UFC normalization and no fetal complications. Of note, one patient required dose escalation up to 5 mg twice weekly to control hypercortisolism by delivery.

Use of high-dose cabergoline in Parkinson's disease has been associated with increased risk for cardiac valvular abnormalities (35). It is recognized that cabergoline doses used for the treatment of pituitary tumors are an order of magnitude lower than those used in Parkinson's disease. Although the cabergoline-associated risk for developing valvular abnormalities has not been specifically studied in patients with CD, treatment of patients with acromegaly or prolactinomas with standard doses, as well as the cumulative dose with long-term follow up, has not been associated with increased valvulopathy (36, 37).

Strong support for use of DA in CD is lacking (32), despite recent data showing clinical benefit (Table 1). If considered, its limitations should be acknowledged, as the clinical response is widely variable and maintaining consistent results is challenging (13). As the majority of studies were performed with cabergoline (Table 1), use of DA in CD should likely be limited to cabergoline.

## Acromegaly

### Preclinical studies

Preclinical data strongly support targeting D2R in acromegaly. Dopamine receptor sites in human growth hormone (GH)-secreting adenomas have been identified (38, 39), and heterodimerization of D2R and somatostatin receptor 5 (SSTR5) forms a Gi protein-linked effector complex (40) that may enhance functional activity, with interaction between the receptor/beta-arrestin complexes of the two receptor families affecting signaling and trafficking of activated receptors (41).

GH-secreting tumors commonly express D2R (9, 10, 42, 43), albeit at levels lower than those in normal pituitary (44). DA treatment of cultured somatotropinomas suppresses GH secretion by 20–25% or more (45–47), while tumors lacking D2R expression show resistance (48). However, as D2R expression positively correlated with *in vitro* but not *in vivo* suppression of GH by quinagolide in 24 somatotroph adenomas (47), the link between D2R expression and treatment response is unclear.

It is possible that D2R isoforms confer differential sensitivity to DA in GH-secreting adenomas. Treatment of GH-secreting murine GH3 cells with nerve growth factor promoted D2R expression, with preferential increase in D2S (29, 30). Exposure of these cells to bromocriptine resulted in decreased cell survival compared with GH3 cells treated with bromocriptine alone (49). Also, bromocriptine treatment of adenovirus D2S-transfected GH3 xenografts in nude mice significantly inhibited tumor growth compared with vector-transfected GH3 cells (50), suggesting that the ratio of D2S to D2L may influence DA inhibition of cell proliferation in GH-secreting tumors.

Co-treating cultures of GH-secreting adenomas with somatostatin receptor ligands (SRL) and DA may be beneficial (51), due, at least in part, to heterogeneous SSTR subtype expression in different tumors and to variable expression levels of D2R and SSTR2/5 (44, 46). Rat pituitary somatomammotrophs strongly coexpressing GH and PRL show high D2R mRNA expression and undergo pronounced GH suppression by bromocriptine (48). However, whether tailoring treatment according to tumor phenotype and/or receptor expression may improve treatment responses is unknown.

### Clinical efficacy

Bromocriptine was the first effective medical therapy for biochemically uncontrolled acromegaly after surgery, but results are modest. Insulin-like growth factor (IGF)-I normalization and tumor size reduction is observed in only 10% and < 20% of patients, respectively, despite a GH reduction in 50% of patients. Quinagolide showed slightly better outcomes, with normalization of GH and IGF-I in 30% of patients (52).

Cabergoline has been more widely studied, and was also proven effective in acromegaly (53). The largest prospective study of cabergoline monotherapy in acromegaly treated 64 patients for 3–40 months and yielded IGF-I normalization in 39%. Patients with GH/PRL-secreting adenomas showed 50% IGF-I normalization, and tumor shrinkage was observed in 13 of 21 patients with macroadenomas (54).

Chanson's meta-analysis of 20 studies showed moderate effects of cabergoline on both tumor size and GH/IGF-I response at doses ranging from 0.3 to 7 mg/week (37, 55). Approximately one-third of patients experienced tumor shrinkage and IGF-I normalization was achieved in 34% of 149 patients treated with cabergoline monotherapy. In five studies encompassing 77 patients uncontrolled with ongoing SRL treatment, the addition of cabergoline normalized IGF-I in 52%. Benefit was mostly limited to patients with moderately increased (1.5 × ULN) baseline IGF-I levels (55). Their updated analysis including 4 additional combination therapy trials reported similar outcomes (37).

Data from the UK Acromegaly Register confirm that DA monotherapy is only moderately successful. Approximately 40% of 1792 patients received DA as monotherapy or in combination with SRL, with cabergoline most commonly delivered in doses of 0.5 mg twice weekly, 1 mg twice weekly, and 0.5 mg daily; normalization of both GH and IGF-I was seen in 26% on DA monotherapy and 20% of patients on combination therapy. As patients treated with combination therapy presumably failed prior monotherapy, lower response rates might be expected, yet success was still more likely in patients with only moderately elevated pretreatment GH levels (56).

Data from the Bulgarian Acromegaly Database of 534 patients showed similar GH/IGF-I responses, but prior radiation therapy predicted better outcomes. Among non-irradiated patients, 16% and 18% of those treated with bromocriptine or cabergoline, respectively, achieved disease control; among those previously irradiated, one-third were controlled after a median of 10 years (57).

Few data are available on the combination of cabergoline and pegvisomant (58–60). Cross-sectional studies show significantly decreased IGF-I with long-term combination treatment in patients with mildly elevated IGF-I despite pegvisomant monotherapy (60). In a prospective trial of this combination, of 24 patients with elevated IGF-I who were treatment-naïve or previously treated with DA or SRL monotherapy, 68% achieved IGF-I normalization on escalating doses of cabergoline (up to 0.5 mg/day) given for 18 weeks plus pegvisomant 10 mg/day for 12 additional weeks. After switching to pegvisomant monotherapy (10 mg/day) for another 12 weeks, only 26% remained controlled (59). Two patients achieved control with cabergoline monotherapy, one with moderately elevated IGF-I (1.3 × ULN) and the other with a GH/PRL-secreting tumor, supporting prior data that these factors likely influence response to DA therapy.

Current guidelines recommend cabergoline as first-line medical therapy for patients with mild IGF-I elevations after surgical adenoma resection (61), and as an add-on to SRL in patients with inadequate biochemical control on SRL monotherapy (61, 62). As bromocriptine is less effective and confers more adverse events than does cabergoline (63, 64), its use in acromegaly is limited.

## Nonfunctioning pituitary tumors

### Preclinical studies

As there are no good laboratory models of NFPT, preclinical data are limited to primary tumor cultures from surgical samples.

Most NFPT express D2R (3, 9, 44), and DA has been shown to reduce gonadotropin secretion (65) and inhibit thymidine incorporation *in vitro* (66). D2R mRNA expression is higher in gonadotroph and null-cell NFPT compared with plurihormonal and silent corticotroph adenomas (67, 68), although D2L/D2S ratios tend to be similar among the histological types (69). NFPT show higher D2R mRNA expression levels compared with somatotroph adenomas, but lower expression compared with normal pituitaries (44) and significantly lower compared with prolactinomas (69). High D2R protein expression levels were found in 26 of 70 NFPT, 26 of 28 prolactinomas, and 18 of 20 GH-secreting adenomas, as well as 31 of 37 FSH-secreting adenomas, 18 of 27 ACTH-secreting adenomas, and 9 of 15 thyrotropinomas (9).

NFPT also express SSTRs (51), but its significance is unclear. Some have shown a positive correlation between mRNA expression of D2R and SSTR2/3 and an inverse correlation with SSTR1/5 (68), while others found no correlation at all (44).

Tumoral D2R abundance may correlate with clinical response to DA treatment (70–74), so scintigraphy using radiolabeled D2R ligands may be useful in identifying tumors likely to respond to DA. In addition, NFPT expressing short D2S show a good response to DA therapy (3, 27). Study of tumor tissue showed D2R mRNA expression in 12 of 18 samples: 6 had only the D2L isoform, 2 had only the D2S isoform, and 4 expressed both. Nine of these patients with residual tumors after surgery were treated with cabergoline for 1 year, and tumor shrinkage was observed in 5, but the small number of patients precluded a firm conclusion regarding correlation between D2S expression and DA treatment response (27).

### Clinical efficacy

Patients with NFPT typically lack serum markers reflecting tumor proliferation, so treatment efficacy is based on tumor size assessment.

In pooled studies through 1992, bromocriptine induced tumor growth stabilization in 76 of 84 NFPT as measured on CT scan in patients with persistent disease (75–82). Nobels et al. saw similar results on CT/MRI in 10 NFPT patients treated with quinagolide for a median of 57 months, with a >10% tumor volume decrease in 3 patients and stable disease in another 3 patients, 2 of whom later saw a decrease in tumor volume on further follow-up (83).

Vieira Neto et al. studied 23 patients with postoperative residual tumors, 9 of whom were treated with cabergoline, starting at an initial dose of 0.5 mg during the first week, and increased by 0.5 mg per week up to 3.0 mg/week, maintained for 6 months. A ≥25% tumor volume reduction was seen on MRI in 6 of 9 patients (median 29%), and D2R expression was detected in all 6 tumors. Most of the untreated patients had stable disease (84).

Greenman et al. reported the largest series of NFPT patients treated with DA (85, 86). Treatment doses were gradually increased up to 10 mg/day of bromocriptine and 2 mg/week of cabergoline. The initial cohort consisted of 33 patients with postoperative residual tumors; 20 received immediate preventive treatment, while 13 were started on DA when tumor growth was noted. A separate cohort of 76 NFPT patients at the same institution served as controls. After a mean 40 months' follow up, 45% saw tumor mass shrinkage and 45% had stable disease with preventive treatment. With secondary treatment, 65% saw tumor shrinkage or stabilization; only 38% had stable disease in the control group (85).

The treatment cohort expanded to 114 patients, and 79 were evaluable: 55 received preventive DA treatment and 24 received treatment upon tumor growth. With preventive treatment, 87% achieved tumor control (38% shrinkage, 49% stabilization); older age and smaller preoperative tumors were independently associated with a better response. With secondary treatment, 58% achieved tumor control (29% shrinkage), while only 47% had stable disease among controls. Median tumor progression-free survival was 6 years for controls and 8.5 years in the secondary treatment group, but was not reached in the preventive treatment group by year 24. The estimated 5-year progression-free survival rate was 88% with preventive treatment, 60% with secondary treatment, and 69% in controls. At 15 years, the rate remained high at 81% with preventive treatment, but fell to 24% with secondary treatment, and to 4% in controls (86). Fewer patients in the preventive treatment group required additional surgery or radiotherapy.

D2R staining was diffusely positive in half of tumors tested across both treatment groups, and short D2S mRNA isoform was more frequently detected. However, none of these features correlated with treatment response (86).

Pooled data on 199 patients in published cases and series found that 28% of DA-treated NFPT patients had reduced tumor size, 64% had stable disease, and only 8.5% experienced tumor growth (87). Updated data including more recent series indicate that 58% show stable disease and 30% show tumor shrinkage (Table 2).

**Table 2:** Response to dopamine agonists in NFPT.

**Study**	***N***	**Mean follow-up (months)**	**Therapy**	**Tumor shrinkage (*n*)**	**Tumor growth (*n*)**	**Stable disease (*n*)**
Wollesen et al. (88)	11	2–33	BRC	9	0	2
Barrow et al. (75)	12	1.5	BRC	6	0	6
Grossman et al. (76)	15	3–36	DA	0	0	15
Verde et al. (78)	20	1–32	BRC	1	0	19
Zarate et al. (79)	7	0.5–12	BRC	0	0	7
Bevan et al. (80)	8	4–12	BRC	0	0	8
van Schaardenburg et al. (81)	25	18	BRC	4	1	20
Ferone et al. (72)	6	6–12	Quinagolide	2	0	4
Kwekkeboom et al. (65)	5	12	Quinagolide	1	0	4
Nobels et al. (83)	10	36–93	Quinagolide	0	6	4
Colao et al. (74)	10		DA	2	0	8
Lohmann et al. (89)	13	12	CAB	7	0	6
Pivonello et al. (27)	9	12	CAB	5	3	1
Garcia et al. (90)	19	6	CAB	6	4	9
Viera Neto et al. (84)	9	6	CAB	6	0	3
Greenman et al. (86)	79	105	CAB	28	17	34
Total	258			77/258 (30%)	31/258 (12%)	150/258 (58%)
Total for BRC	83			20/83 (24%)	1/83 (1%)	62/83 (75%)
Total for CAB	154			54/154 (35%)	24/154 (16%)	76/154 (49%)
Total for Quinagolide	21			3/21 (14%)	6/21 (29%)	12/21 (57%)

*BRC, bromocriptine; CAB, cabergoline; DA, dopamine agonist; NFPT, nonfunctioning pituitary tumor*.

DA therapy is not routinely recommended for patients with persistent/recurrent NFPT due to insufficient evidence (91, 92). The emergence of recent data (86) may prompt a revisiting of this question, but further study is still required to identify the optimal use of this treatment in different clinical scenarios.

## Conclusions

DA are the first-line treatment for prolactinomas. The presence of D2R in all pituitary cell types, data from *in vitro* studies, and clinical data suggest DA may also be an effective primary or second-line treatment for some patients with CD, and as second-line therapy in selected patients with persistent/recurrent acromegaly or NFPT.

## Author contributions

OC and YG conceived and designed the project. OC collected and analyzed the evidence and prepared the draft manuscript. OC and YG contributed to manuscript revisions and approved the submitted version.

### Conflict of interest statement

The authors declare that the research was conducted in the absence of any commercial or financial relationships that could be construed as a potential conflict of interest.
